# The genus *Gymnetron* from China with description of pre-imaginal stages of *G. miyoshii*, *G. auliense* and *G. vittipenne* (Coleoptera,Curculionidae)

**DOI:** 10.3897/zookeys.534.5967

**Published:** 2015-11-11

**Authors:** Chunyan Jiang, Runzhi Zhang

**Affiliations:** 1 CAS Key Laboratory of Zoological Systematics and Evolution, Institute of Zoology, Chinese Academy of Sciences, Beijing 100101, P. R. China; 2 University of Chinese Academy of Sciences, Beijing 100049, P. R. China; 3 State Key Laboratory of Integrated Management of Pest Insects and Rodents, Institute of Zoology, Chinese Academy of Sciences, Beijing 100101, China

**Keywords:** *Gymnetron*, larva, pupa, Mecinini, Curculioninae, gallmakers, China, protective chemicals, environmental stress

## Abstract

There are four species of *Gymnetron* in China recorded to date including *Gymnetron miyoshii* Miyoshi, 1922, *Gymnetron villosipenne* Roelofs, 1875, *Gymnetron auliense* Reitter, 1907 and *Gymnetron vittipenne* Marseul, 1876, of which the last two are new country records. The pre-imaginal stages including eggs, mature larvae and pupae of *Gymnetron miyoshii*, *Gymnetron auliense* and *Gymnetron vittipenne* are described and illustrated. In addition, their diagnostic characters (larvae and pupae) are discussed and differentiated, and notes on some of their biological parameters are provided. Potential ecological impacts between *Gymnetron* weevils and their host *Veronica* spp. also are provided.

## Introduction

*Gymnetron* Schoenherr, 1825 belongs to the tribe Mecinini Gistel, 1848 in the subfamily Curculioninae Latreille, 1802 (Alonso-Zarazaga et al. 1999, Caldara 2001). These weevils are small, distinguished from other Mecinini by the following features taken together: prosternum without median sulcus; elytral margin covering a large portion of the pygidium; elytral striae 3 and 8 joined at apex (Caldara 2008). This genus is widely distributed in the Palaearctic and Afrotropical regions (Alonso-Zarazaga et al. 1999, Caldara 2001, 2003, 2008); distribution in China of *Gymnetron miyoshii* Miyoshi, 1922 and *Gymnetron villosipenne* Roelofs, 1875 is recorded by Caldara (2008). The Palaearctic species of *Gymnetron* live on *Veronica* (Caldara 2008), currently included in the Plantaginaceae (Stevens, 2012), while those in the Afrotropical region (Caldara 2003) appear to live on various genera of Scrophulariaceae belonging to the tribes Hemimerideae and Selagineae, *Buddleja* of the Buddlejeae and *Anastrabe* of the Stilbaceae, both families very closely related to Plantaginaceae (Stevens, 2012). The immatures of some species of *Gymnetron* have been studied previously, but without detailed descriptions (van Emden 1938, Scherf 1964, Anderson 1973, May 1993).

The aim of the present study is to describe for the first time all developmental stages of three species of *Gymnetron* living in China in order to provide further characters for the identification of these taxa.

## Materials and methods

Six last instar larvae and ten pupae of *Gymnetron miyoshii*, five last instar larvae and one pupa of *Gymnetron villosipenne*, and ten last instar larvae and ten pupae of *Gymnetron vittipenne* were examined. Descriptions were made and photographs of pupae were taken with a Canon-5D camera mounted on a Nikon SMZ 1500 microscope. Images of adults were photographed with a CCD Qimagine MicroPublisher 5.0 RTV mounted on a Zeiss SteREO Discovery. V12 microscope; Microscopic slides were studied with a Leica DM 2500 microscope and photos were taken with a Nikon CoolPix 5400. Drawings were made from the original photographs by using the software Adobe Illustrator CS6; photos in the field were taken with Canon G15 and 5D Mark II cameras.

Nomenclature of the larval chaetotaxy mainly follows van Emden (1938), May (1993, 1994), Marvaldi (1999) and Wang et al. (2013), and that of the pupa mainly follows Gosik (2010). The dissecting method used follows May (1979, 1994). Indistinct structures were pigmented with “Chlorazol Black E” for further examination. In pupae, *msns* and *mtns* are used as abbreviations of mesonotal setae and metanotal setae, respectively. As *msns* and *mtns* are different among the three weevils species examined, these can be added as special diagnostic characters in *Gymnetron*; in order to differentiate from alar setae and apical setae of the pupa, *as* and *asp* are used, respectively. In the descriptions, setae of the thorax and abdomen are described for one side only.

After description, all larvae and pupae were mounted using nail polish, a mixture of butyl acetate, ethyl acetate, multipolymer of adipic acid, neopentyl glycol, trimellitic acid and acetyl tributyl citrate. All slides remain together with the adult specimens in the museum of the Institute of Zoology, Chinese Academy of Sciences.

## Descriptions

### Gymnetron miyoshii

Taxon classificationAnimaliaColeopteraCurculionidae

Miyoshi, 1922

Gymnetron miyoshii Miyoshi, 1922: 253
Gymnetron villosulum var. orientale Voss, 1955: 139

#### Description.

**Figures 1–8.**
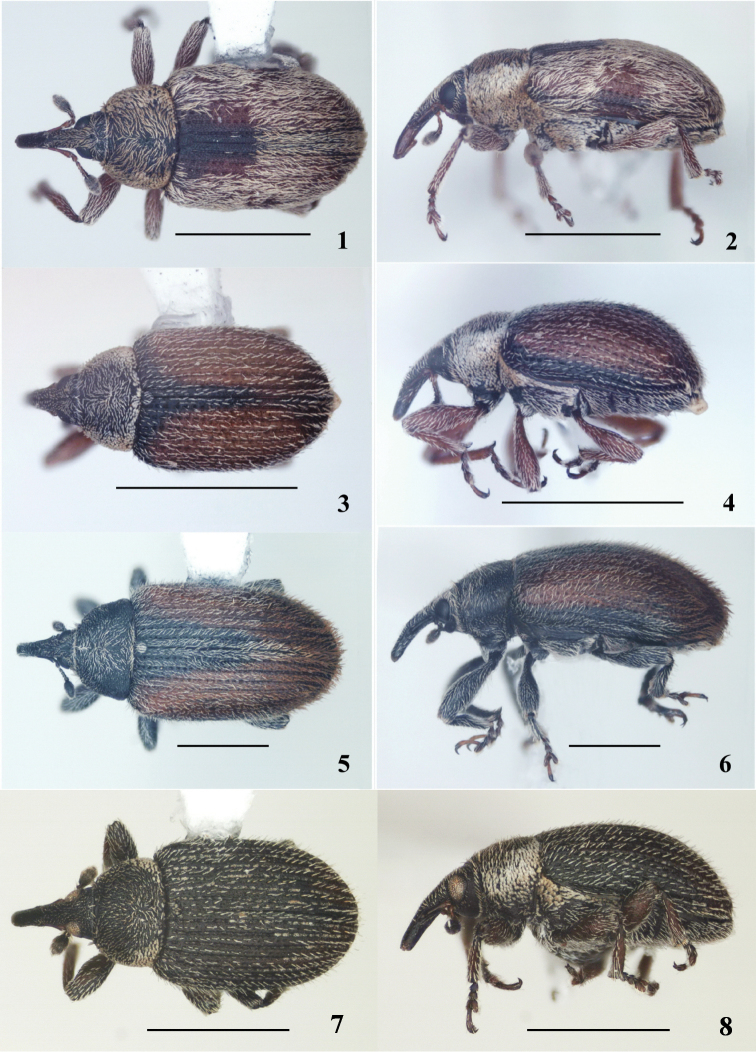
Adults of *Gymnetron*. **1–2** *Gymnetron miyoshii* **3–4** *Gymnetron auliense* **5–6** *Gymnetron vittipenne* **7–8** *Gymnetron villosipenne*. Scale bars: **1–8** 1 mm.

**Adult** (Figures 1–2): sides of pronotum in part, mesothoracic epimera, metasternum and urosternite one covered with broad scales; elytral vestiture forming indistinct spots; rostrum in lateral view slightly curved, in female nearly of same width from base to apex (Caldara 2008).

**Egg**: oval, yellowish, nearly pellucid. Measurements (mm): diameter: 0.11–0.12 (n = 3), length: 0.28 (n = 3).

**Figures 9–10.**
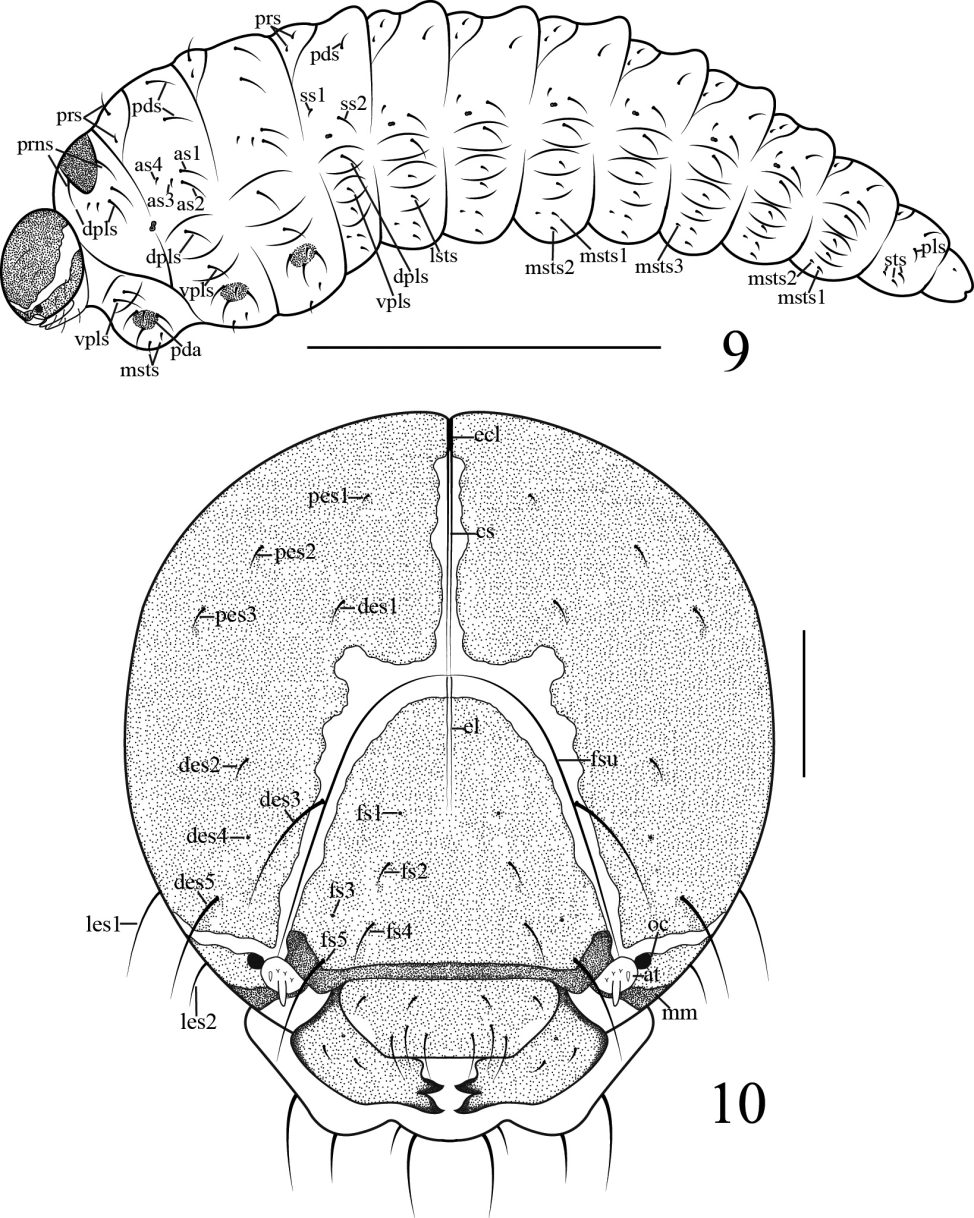
Larva of *Gymnetron miyoshii*. **9** Mature larva, lateral view **10** Head, dorsal view. Scale bars: 1 mm (**9**), 0.1 mm (**10**).

**Mature larva** (Figure 9): Measurements (mm): body length: 3.25–3.90 (n = 2), width: 1.08–1.30 (n = 2); capsule length (in front view): 0.48–0.50 (n = 4), width: 0.38–0.43 (n = 4); body slender and weakly curved, yellowish, subcylindrical, widest at thorax in lateral view, attenuate posteriorly; head brown with pale stripes at sides and along ecdysial line; cuticle minutely spiculate; pronotum partly pigmented and sclerotized; body segments with minute setae, pedal lobes in conspicuous pigmented knobs.

**Figures 11–15.**
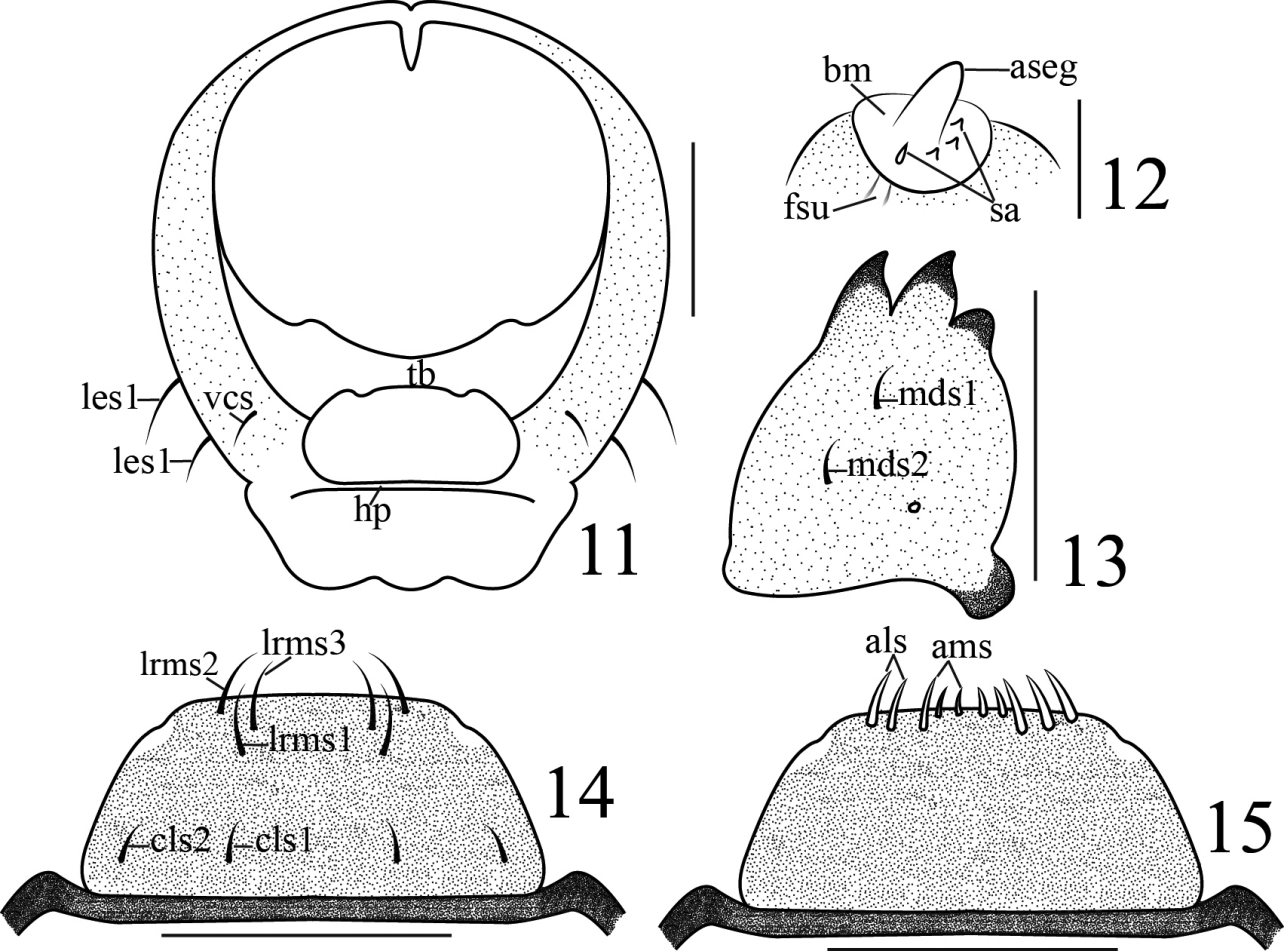
Larva of *Gymnetron miyoshii*. **11** Head, ventral view **12** Antenna **13** Mandible **14** Labrum and clypeus **15** Epipharynx. Scale bars: 0.1 mm (**11**), 0.025 mm (**12**), 0.1 mm (**13–15**).

**Head** (Figures 10–11): free, circular in outline, broader than long, broadest at middle; cranial suture undivided, wide, half length of head; frontal suture distinct, not extending to mandibular membrane; endocarinal line short, no more than half as long as frons; frons with three pairs of *fs*, *fs1* and *fs3* reduced to basal sensilla, *fs*5 longest, laterally positioned on epistoma close to antenna, *fs4* located near epistoma, half as long as *fs5*, *fs2* located in the middle of frons, half as long as *fs4*; dorsal epicranium with four pairs of *des*, *des4* reduced to a basal sensillum, *des3* longest, located on frontal line, *des5* lightly shorter than *des3*, *des2* approximately one quarter as long as *des3*, *des1* slightly shorter than *des2*; epicranium with two pairs of *les*, *les1* long, *les2* short, about half as long as *les1*; posterior epicranium with three pairs of *pes*, *pes1* minute, *pes2* equally as long as *pes3*; ventral epicranium with one pair of *ves*, minute; postoccipital condyles indistinct, hypopharyngeal bracon distinct; tentorial bridge narrow, with two small but moderately acute anterior projections and two large, obtuse-angled posterior projections; clypeus transverse, fused to labrum, bearing two pairs of *cls*, *cls1* nearly same length as *cls2*, located in one line, parallel to clypeus, sensilla absent; antenna (Figure 12) one segment, sensory appendage nearly twice as long as wide, circular in cross-section, contiguous with frontal suture, with one conical and three minute sensilla; ocellus present, not projecting, located below stripe at side, externally close to antenna.

**Figures 16–17.**
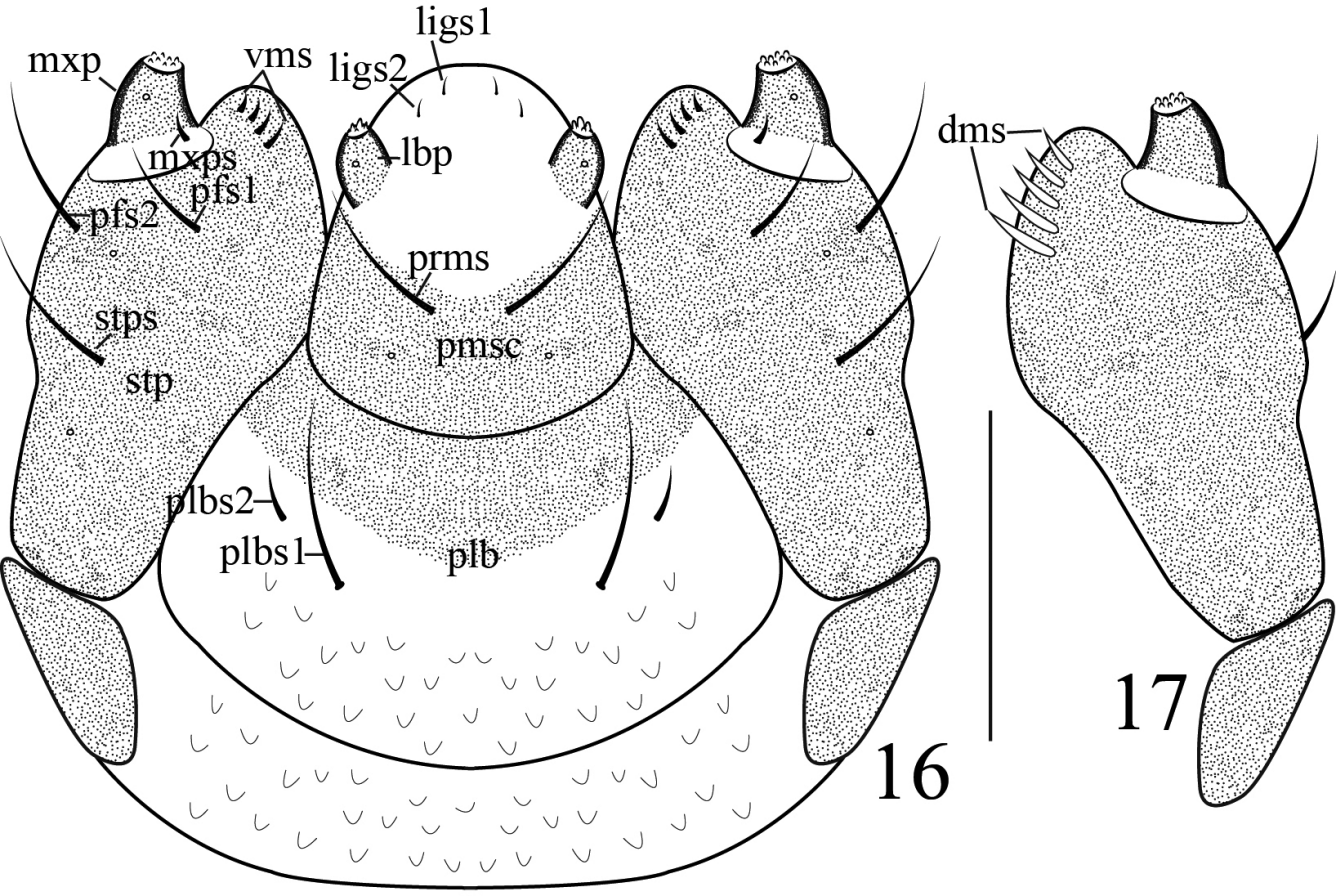
Larva of *Gymnetron miyoshii*. **16** Labium, ventral **17** Maxilla, dorsal. Scale bars: 0.1 mm.

**Mouthparts** (Figures 13–17): mandibles (Figure 13) symmetric, incisor section with two apical teeth and rounded flange posterior to dorsal tooth, molar section with two *mds*, *mds1* nearly same length as *mds2*, sensilla distinct; labrum (Figure 14) transverse, fused to clypeus, nearly completely sclerotized, with three pairs of *lrms*, *lrms2* slightly shorter than *lrms1*, both centrally localized, *lrms3* same as *lrms1*, close to distal margin of labrum; epipharynx (Figure 15) with all epipharyngeal setae stout and apically rounded, with two pairs of *als*, three pairs of *ams*, epipharyngeal sensilla, *mes* and labral rods (tormae) absent. Labium (Figure 16) membranous excepting the premental sclerite, labial palpus with one segment, slightly longer than wide, apex of palpus flattened with dense short irregular spiculate setae, and one sensillum; premental sclerite (*Pmsc*) distinctly posteriorly and laterally dilated, U-shaped, with one pair of sensilla and one pair of long *prms*. Ligula with two pairs of tiny *ligs*, *ligs1* as long as *ligs2*. Postlabium (*plb*) partly sclerotized, with two pairs of *plbs* at membranous area, *plbs1* long, *plbs2* short, one quarter long as *plbs1*; membranous area sparsely and finely asperate. Maxillae with maxillary palpus (*mxp*) (Figures 16–17) two-segmented, basal segment with one tiny mxps, accessory appendage absent; distal segment sclerotized, apex flattened with dense short irregular spiculate setae, one sensillum; mala with five dorsal robust *dms*, *dms1–5* gradually shorter, with four shorter, more acute *vms.* Stipes bearing one *stps*, two *pfs* and two sensilla, *stps* strong and long, submedian on venter of base. *Pfs1* a little shorter, located near mala, one third as long as *pfs2*, *pfs2* submedian on venter of base, cardo completely divided from stipes.

**Figures 18–19.**
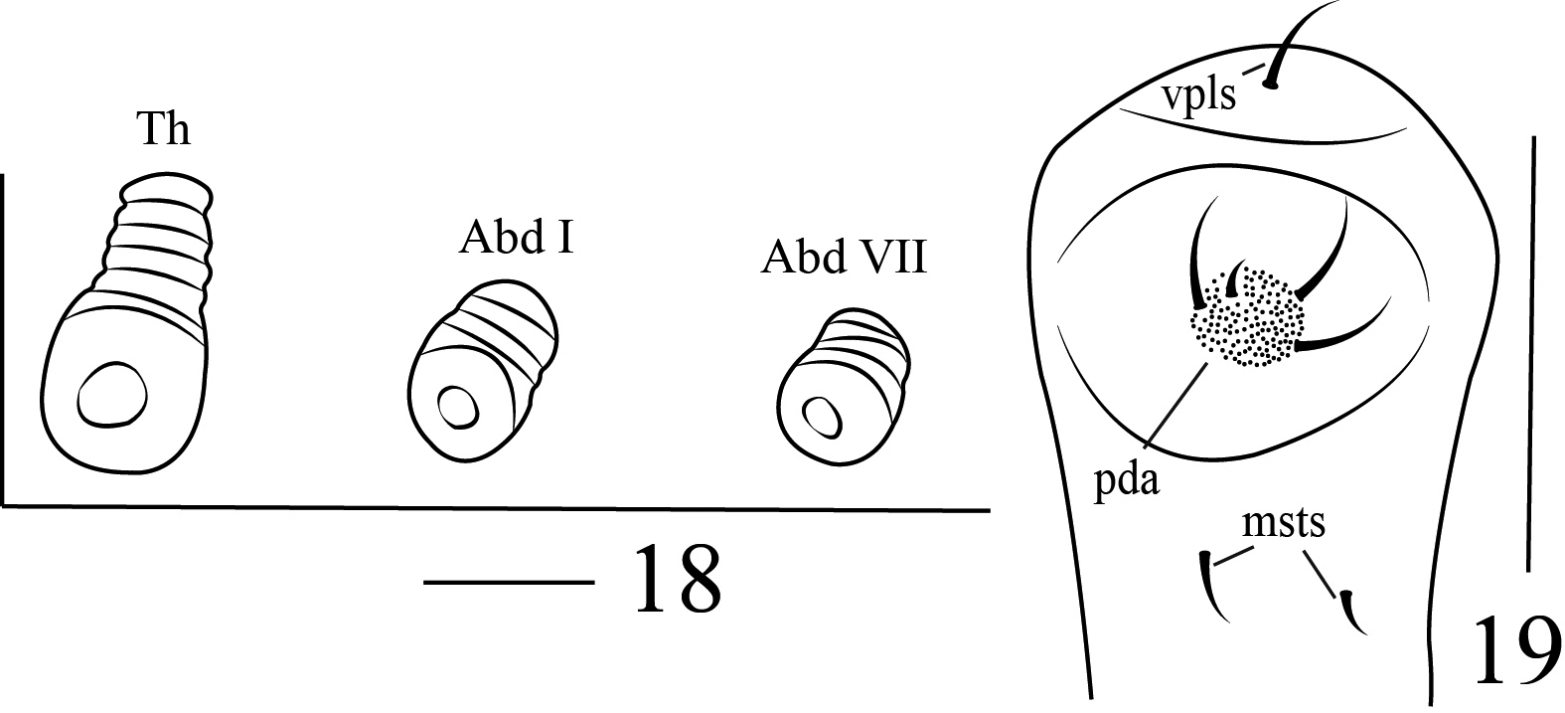
Larva of *Gymnetron miyoshii*. **18** Spiracles, showing alignment **19** Pedal and mediosternal setae. Scales bars: 0.0325 mm (**18**), 0.05 mm (**19**).

**Thorax** (Figure 9): pronotal shield partly pigmented and sclerotized on pale smooth plate. Pronotum with two setae on sclerotized area, dorsopleurum with four *dpls*; spiracle (Figure 18) intersegmental between pro- and mesothorax, bicameral, air-tube subequal to diameter of circular peritreme; ventropleurum with two *vpls*; pedal area (Figure 19) distinct, pedal lobe present, one-segmented, slightly convex, with four setae; mediosternum with two *msts*. Mesonotum with two folds (prodorsum and postdorsum), prodorsum with two *prs*, postdorsum with two *pds*, two setae transversally aligned; alar area with four *as*, two long, two short; dorsopleurum with one *dpls*, ventropleurum with one *vpls*; setae of pedal area and mediosternum same as prothoracic. Metanotum same as mesonotum.

**Abdomen** (Figure 9): with spiracles on segments I-VII, size similar, all anterolateral and unicameral, each with single annulated air-tube, pointing posteriad, subequal to diameter of circular peritreme. Abdominal segments I-VII with tergites with two folds, prodorsum with two *prs*, *prs1* longer than *prs2*, postdorsum with soft protuberance posteriorly, with one *pds*, all setae shorter than thoracic setae; spiracular area with two *ss*, *ss1* short, one quarter as long as *ss2*; dorsopleurum with one *dpls*, ventropleurum with one *vpls*, laterosternum with one *lsts*, mediosternum with three *msts*, except *msts3* in front of *msts1*, other five setae short and transversally aligned. Abdominal segment VIII with tergite with two folds, prodorsum with one *prs*, postdorsum with one *pds*; spiracular area with two *ss*, *ss1* short, one quarter as long as *ss2*; dorsopleurum with one *dpls*, ventropleurum with one *vpls*, laterosternum with one *lsts*, mediosternum with one *msts*, except *msts2* in front of *msts1*, other four setae short and transversally aligned. Abdominal segment IX with tergite with two folds, prodorsum with one *prs*, postdorsum with one *pds*; pleurum with one *pls*, sternum with three *sts.* Abdominal segment X with one tiny seta, anus transverse cleft.

**Figures 20–22.**
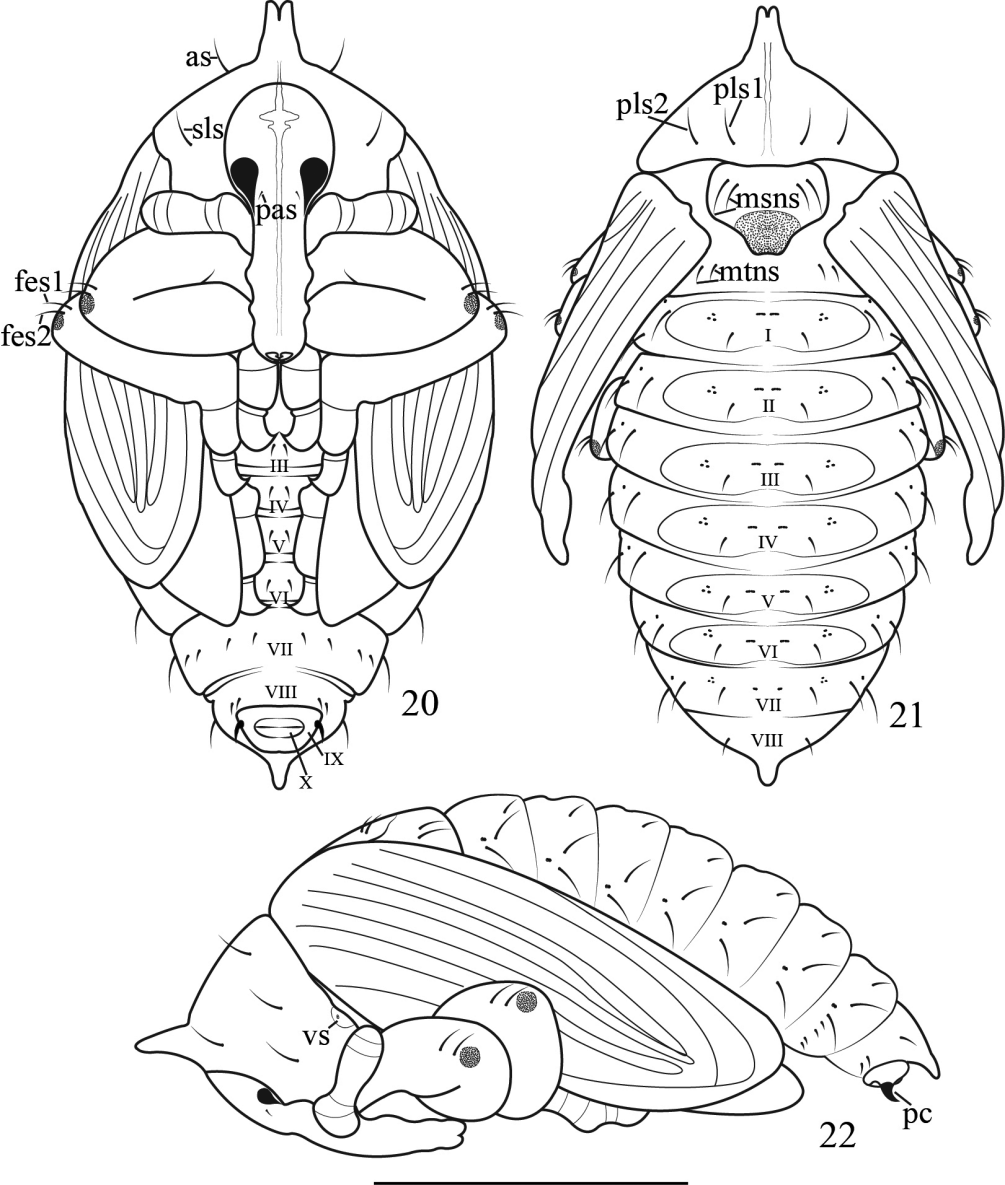
Pupa of *Gymnetron miyoshii*. **20** Ventral view **21** Dorsal view **22** Lateral view. Scales bars: 1 mm.

**Pupa** (Figures 20–22): Measurements (mm): length: 2.65–3.00 (n = 4), width: 1.00–1.60 (n = 4), height: 1.25–1.50 (n = 4). **General appearance**: Theca yellow, grayish at apex of antennae, rostrum, legs, wings, elytra, anus and apex and base of ventrites. Setae greatly reduced in number. Ventrites III-X visible in ventral view, tergum I-VIII visible in dorsal view. **Head**: yellow-gray with one yellow stripe along middle, with one pair of *pas*, situated at middle margin of eyes; eyes large, one third of length of head, not projecting; rostrum long, twice as long as wide, mesorostrum visibly dilated, mandibular theca weakly projecting, setae absent; antennae applied against prosternum and apically extending to propleurum, subparallel to profemur. **Thorax**: prothorax bearing one median, lightly pigmented tubercle, apically shallowly bifurcate, with a spiracle between pronotum and mesonotum, but lacking air-tube; pronotum with one *as* and one *sls* in ventral view, two *pls* in dorsal view, *as*, *sls* and *pls* subequal, strong and long, *pls1* and *pls 2* positioned in one transversal row; mesonotum with two *msns* on scutellum; metanotum bearing two *mtns* near hind margins, half shorter in length than pronotal setae. **Legs**: pro-, meso- and metafemora apically bearing two slightly outcurved *fes*, *fes 1* as long as *fes 2*, apex with grey circular pigmented area. **Abdomen**: segments I-VII with tergite bearing one seta, with transversely oval impression, submedian small transverse macula and lateral maculae, segment VII lacking impression. Spiracles present on segments I-VII, spiracular area with one *ss*, dorsopleurum with one *dpls*, ventropleurum with one *vpls*, laterosternum with one *lsts*, mediosternum with two *msts*; segment VIII with tergite bearing one fleshy, pigmented, apically narrowing rounded process, with two seta on tergite, sternum with two setae; segment IX with sclerotized, bifurcate, elongate and slightly curved outward pseudocerci, subterminally positioned at ventral abdominal segment IX, invisible in dorsal view; segment X with anus transverse cleft, subterminal, invisible in dorsal view.

#### Specimen examined.

**CHINA: Hunan**: Food and Drug Administration of Changsha (28°11.85'N; 113°0.36'E, 43m), 18-VIII-1975, *Veronica anagallis-aquatica* L.(8); same except: Hunan Medical University (28°18.05'N; 112°52.14'E, 48m), VII-1979, *Veronica* spp.(3); Institute of Plant Protection of Hunan (28°12.01'N; 113°04.86'E, 45m), 1973, *Veronica anagallis-aquatica* L. (2); 1975 (6); 24-V-1976, *Veronica anagallis-aquatica* L., leg Yangchang Zhao (10); 21-V-1976, *Veronica anagallis-aquatica* L., leg Qiong Zhu (2); 16-V-1976, *Veronica anagallis-aquatica* L. (5). **Inner Mongolia**: Hohhot (40°49.28'N; 111°38.82'E, 1289m), 15-VI-1965, *Veronica undulata* Wall., leg Hongchang Li (5); Molidawanqi, Hulun Buir (48°28.53'N; 124°30.18'E, 335m), 23-VII-1940 (1). **Hebei**: Manzuxiang, Dongling, Zunhua, Tangshan, (40°10.90'N; 117°54.49'E, 120m), 8-VII-1963, *Veronica* spp., leg Wenzhen Ma (1). **Beijing**: Badaling, (40°20.49'N; 115°58.88'E, 669), 20-VI-1963, *Veronica anagallis-aquatica* L., leg Tiesheng Li (9); Sanpu, Yanqing (40°19.65'N; 116°02.18'E, 593m), 28-VII-1980, *Veronica undulata* Wall. (18); 1980-VI-29, *Veronica undulata* Wall., leg Shengqiao Jiang (10); 7-VII-1980, *Veronica anagallis-aquatica* L., leg Subai Liao (29); Beizhaicun, Qiaozizhen (40°19.77'N; 116°33.34'E, 73m), 1-VI-2013, *Veronica anagallis-aquatica* L., leg Chunyan Jiang (26); 24-V-2014, *Veronica anagallis-aquatica* L., leg Chunyan Jiang (4); 14-VI-2014, leg Chunyan Jiang (5 eggs, 13 larvae, 6 adults). **Jiangsu**: Yinqiao, Suyang, Changzhou (31°25.94'N; 119°29.73'E, 8m), 5-VI-1981 (1); Wujin (31°40.16'N; 119°55.93'E, 4m), 12-V-1955 (1); Hangzhou (30°15.33'N; 120°12.50'E, 6m), 1982, leg Guangsheng Li (1). **Heilongjiang**: Harbin (45°45.94'N; 126°38.70'E, 116m), 18-V-1945 (1).

#### Biological notes.

*Veronica anagallis-aquatica* L. has been collected with galls on 14-VI-2014 which have been reared in the laboratory. Fifteen pupae were found on 21-VI-2014.

### Gymnetron auliense

Taxon classificationAnimaliaColeopteraCurculionidae

Reitter, 1907

Gymnetron melinum var. auliense Reitter, 1907: 30.
Gymnetron auliense : Caldara 2008: 38.

#### Description.

**Adult** (Figures 3–4): Sides of pronotum covered with dense, imbricate, broad scales; elytral integument reddish and black, rarely completely black, interstriae covered with recumbent to suberect seta-like scales arranged in two-three rows; rostrum moderately robust, scarcely sexually dimorphic, in lateral view moderately curved, weakly narrowed at apical third (Caldara 2008).

**Egg**: unknown.

**Figure 23–24.**
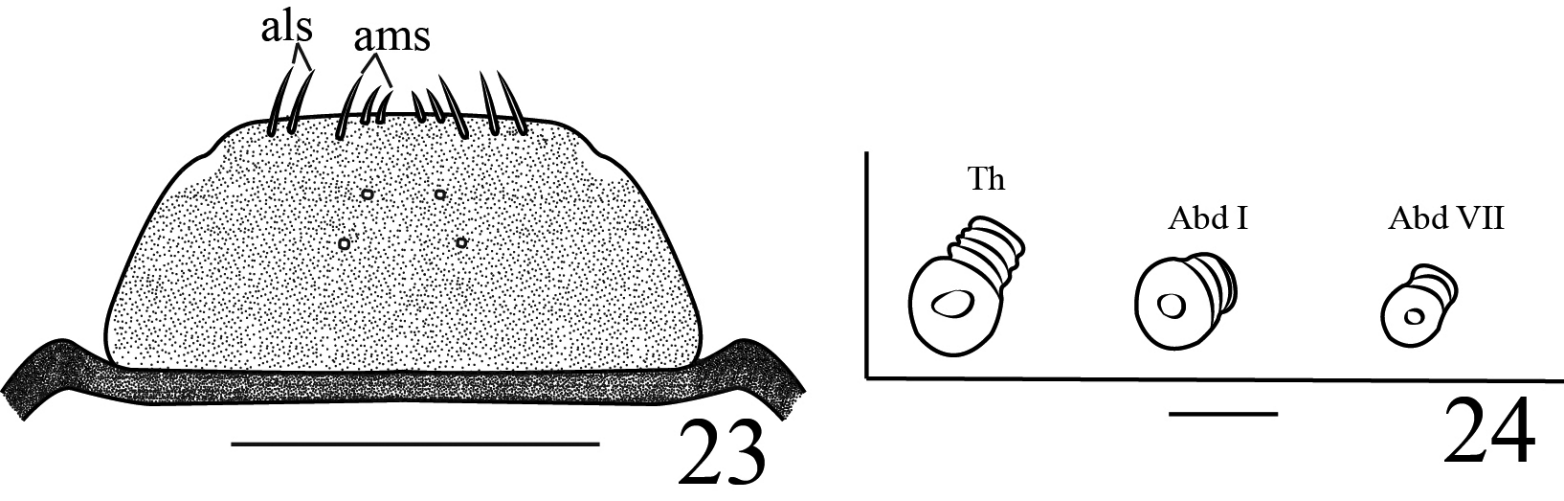
Larva of *Gymnetron auliense*. **23** Epipharynx **24** Spiracles, showing alignment. Scales bars: 0.1 mm (**23**), 0.025 mm (**24**).

**Figure 25–26.**
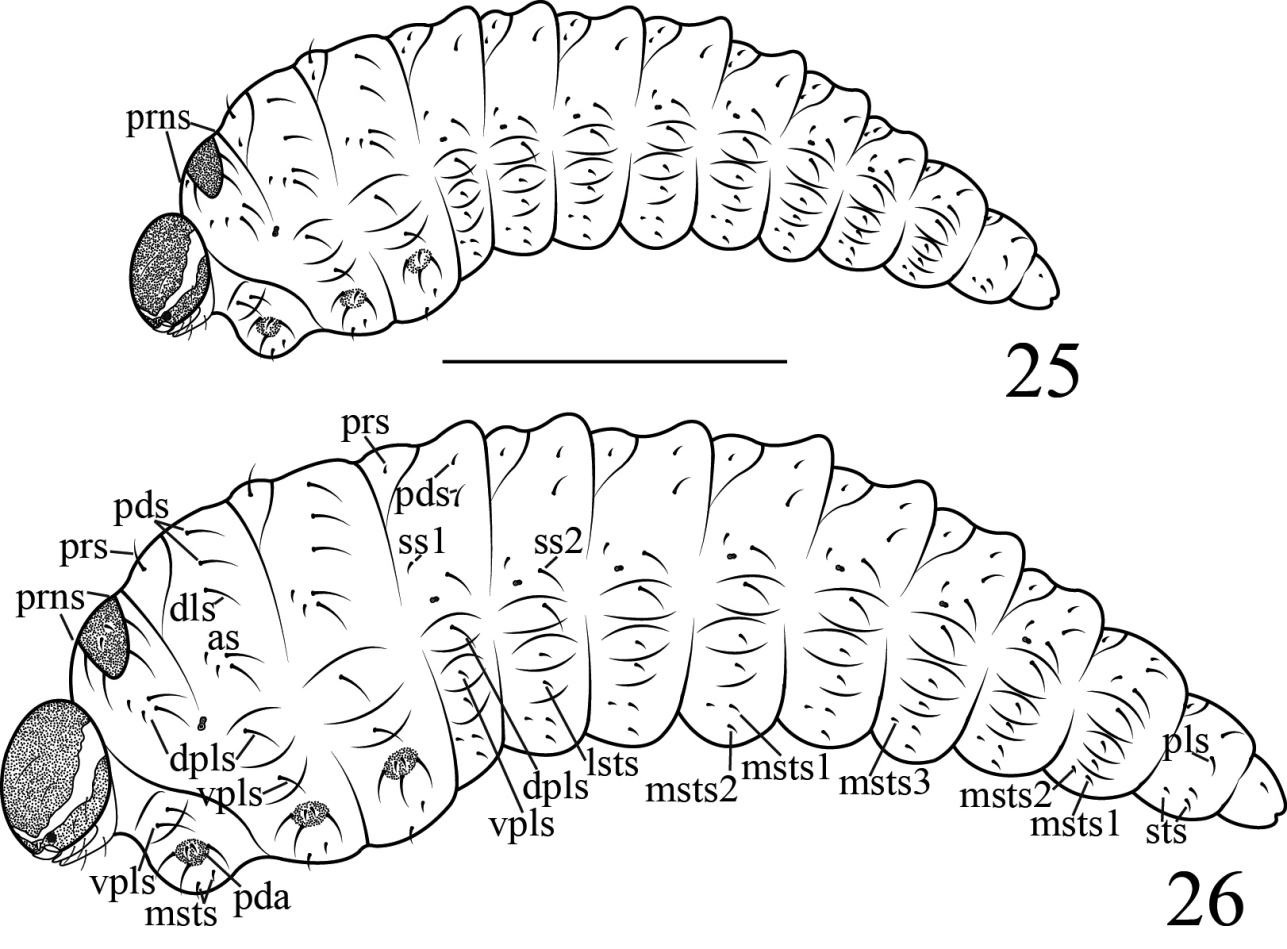
Two larvae. **25** Larva of *Gymnetron Auliense* **26** Larva of *Gymnetron vittipenne*. Scales bars: 1 mm.

**Mature larva**: measurements (mm): body length: 2.40–2.50 (n = 4), width: 1.20–1.45 (n = 4); capsule length (in dorsal view): 0.50–0.55 (n = 3), width: 0.46–0.50 (n = 3). It differs from *Gymnetron miyoshii* by: **Mouthparts**: epipharynx (Figure 23) with two pairs of sensilla, all epipharyngeal setae distinctly slender. **Thorax** (Figure 25): pronotum with four *pns*, dorsopleurum with four *dpls*. Spiracle bicameral, air-tube equal or shorter than diameter of circular peritreme. **Abdomen**: with seven spiracles, size similar, all anterolateral and unicameral, each with single annulated air-tube, shorter to diameter of circular peritreme.

**Pupa**: Measurements (mm): length: 2.80 (n = 1); width: 1.00 (n = 1).

Mesonotum with three pairs of *msns* on scutellum; Pro-, meso- and metafemora of legs apically bearing one slightly outcurved *fes*, apex with grey circular pigmented area.

#### Specimen examined.

**CHINA**: **Xinjiang**: Kaerjiaocun, Jimunai (47°13.03'N; 86°24.12'E, 1224m), 3-VII- 2014, leg Chunyan Jiang (6).

#### Biological notes.

After collecting specimens of *Veronica oxycarpa* Boiss. on 3-VII- 2014, for rearing in the laboratory, 5 larvae were obtained on 9-VII-2014 and 1 pupa on 12-VII-2014.

#### Remarks.

This species has been recorded from Kazakhstan, Kyrgyzstan, Tajikistan, Turkmenistan and Uzbekistan. This species is a new record for China.

### Gymnetron vittipenne

Taxon classificationAnimaliaColeopteraCurculionidae

Marseul, 1876

Gymnetron vittipenne Marseul, 1876: 383.
Gymnetron apicale Faust 1885: 187.
Gymnetron vittipenne var. suturiferum Reitter 1907: 32.

#### Description.

**Adult** (Figures 5–6): Sides of pronotum covered with dense, imbricated, broad scales; uncus of metatibiae strongly enlarged at apex in male; first tarsal segment on venter covered with very dense and long setae in male; ductus of spermatheca sclerotized at base near insertion with spermatheca. Elytra parallel-sided, with reddish and black integument covered with moderately dense, recumbent to erect, seta-like scales arranged in three very irregular rows on each interstria; rostrum in lateral view slightly curved, angulate along dorsal margin at antennal insertion and weakly narrowed at apical third in male, strongly curved, cylindrical and of same length from base to apex in female (Caldara 2008).

**Egg**: unknown.

**Mature larva**: measurements (mm): body length: 5.00–5.20 (n = 2), width: 1.60–2.00 (n = 2); capsule length (in dorsal view): 0.70–0.86 (n = 4), width: 0.57–0.68 (n = 4).

**Figures 27–34.**
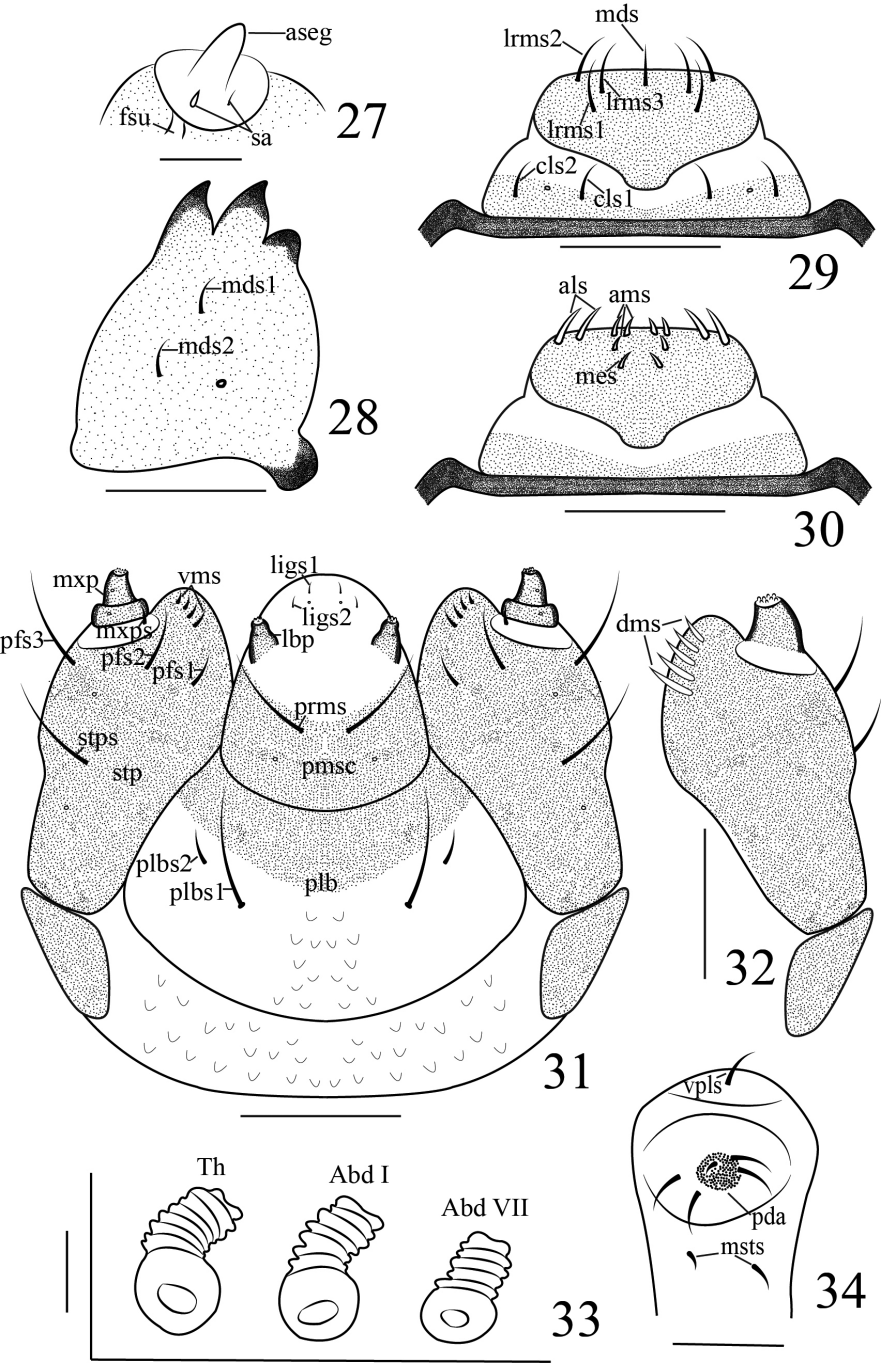
Larva of *Gymnetron vittipenne*. **27** Antenna **28** Mandible **29** Labrum and clypeus **30** Epipharynx **31** Labium, ventral **32** Maxilla, dorsal **33** Spiracles, showing alignment **34** Pedal area. Scales bars: 0.025 mm (**27, 33**), 0.1 mm (**28–32**), 0.05 mm (**34**).

It differs from *Gymnetron miyoshii* by: **General appearance** (Figure 26): size greater. **Head**: Size greater, endocarinal line long, more than half as long as frons; hypopharingeal bracon distinct; clypeus transverse, bearing two pairs of *cls*, *cls1* nearly same length as *cls2*, located in one line, parallel to clypeus, sensilla distinct; antenna (Figure 27) with one segment, contiguous with frontal suture, with one spinose and one tiny seta-like sensilla. **Mouthparts** (Figures 28–32): labrum (Figure 29) transverse, partly sclerotic, anterior margin nearly straight, posterior margin weakly extended medially into clypeal zone, with three pairs of *lrms*, *lrms2* a bit shorter than *lrms1*, both centrally localized, *lrms3* same length as *lrms1*, close to distal margin of labrum, with one *mds*, subequal to *lrms2*; epipharynx (Figure 30) with two pairs of *als*, three pairs of *ams*, one pair of *mes*, sensilla absent. All epipharyngeal setae stout, short and apically rounded; labium (Figure 31) membranous except sclerotized area. Labial palpus with one segment, longer than wide distinctly, with one pair of sensilla, apically flattened with dense crenulate setae. Premental sclerite (*Pmsc*) distinctly posteriorly and laterally dilated, U-shaped, with one pair of sensilla and one pair of long *prms*. Ligulate area with two pairs of tiny *lgs*, *lgs1* same lengtgh as *lgs2*, with one pair of sensilla. Postlabium partly sclerotized, with two pairs of *plbs* at membranous area, *plbs1* long, *plbs2* short, one quarter as long as *plbs1*.; Maxillae with maxillary palpus (*mxp*) (Figures 31–32) two segmented, basal segment distinctly wider than long, with one pair of sensilla and one pair of short *mxps*, accessory appendage absent. Apical segment longer than wide, with one pair of sensilla, apically flattened with dense short irregular speculate setae. Mala with five robust *dms*, *dms1–5* gradually shorter than the former one and four thin *vms.* Stipes bearing one *sts*, three palpiferal *pfs* and two sensilla, *sts* strong and long, basally medioventral, *pfs1* short, located near mala, one third as long as *pfs2*, *pfs2* basally medioventral, same length *pfs3*, *pfs3* lateroventral. Cardo completely divided from stipes. **Thorax** (Figure 26): Pronotum with six *pns.* Spiracle (Figure 33) bicameral, air-tube distinctly longer than diameter of circular peritreme, pointing basad. Pedal area (Figure 34) distinct, with five setae; Mesonotum with two folds, prodorsum with one *prs.* Postdorsum with two *pds*, one *dls* transversally aligned. Pedal area same as prothoracic; Metanotum same as mesonotum. **Abdomen** (Figure 26): with seven spiracles on segments I-VII, size similar, all anterolateral and unicameral, each with single annulated air-tube, distinctly longer than diameter of circular peritreme, pointing basad. **Abd I-VII**: tergites with two folds, prodorsum with one tiny *prs*, postdorsum with soft protuberance posteriorly, with two *pds*, *pds1* short, half as long as *pds2*. All setae shorter than thoracic setae; **Abd VIII**: *pds1* short, half as long as *pds2*.

**Figures 35–37.**
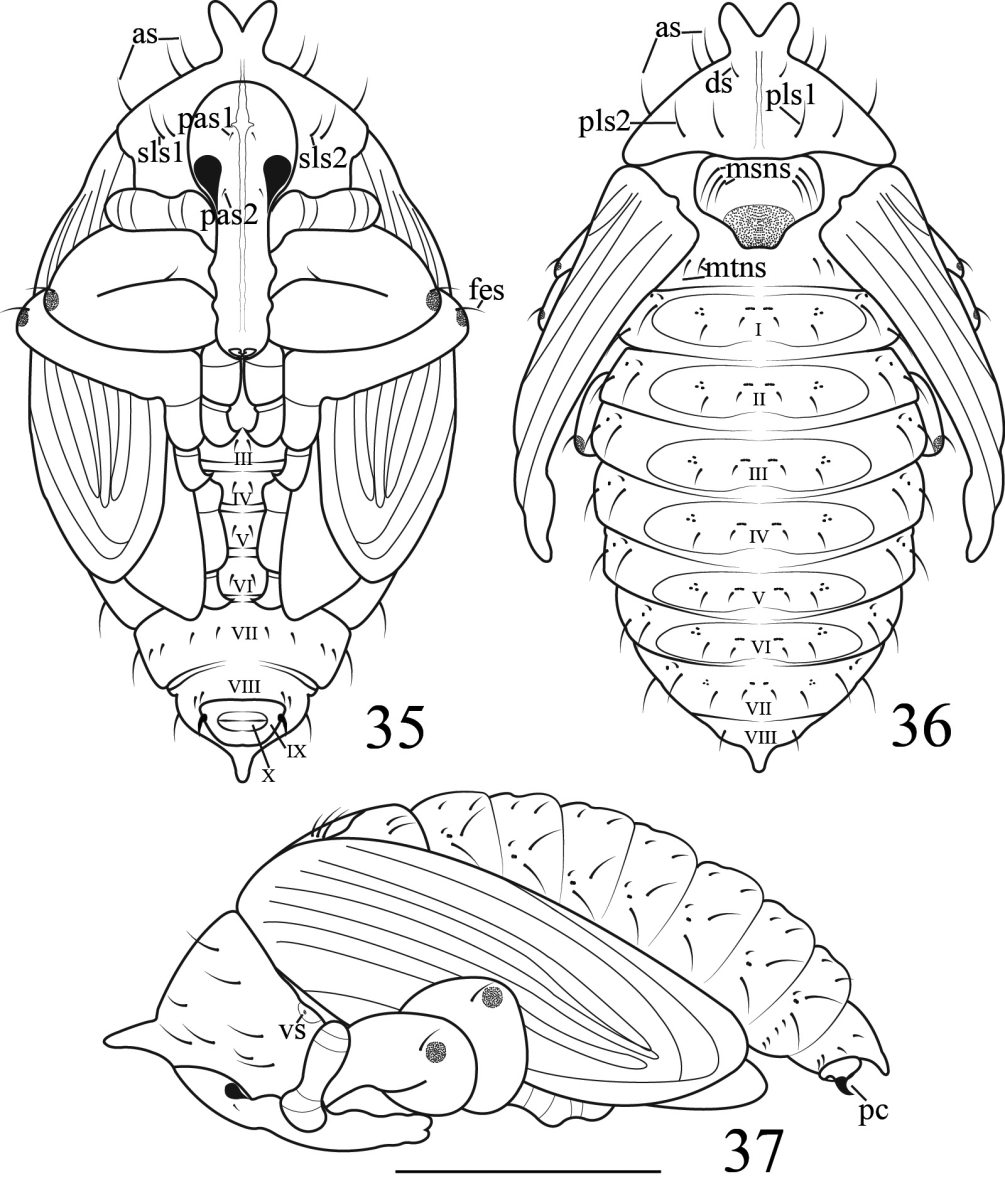
Pupa of *Gymnetron vittipenne*. **35** Ventral view **36** Dorsal view **37** Lateral view. Scales bars: 1 mm.

**Pupa** (Figures 35–37): measurements (mm): length: 4.75–4.85 (n = 4), width: 1.60–2.00 (n = 4).

It differs from *Gymnetron miyoshii* by: **General appearance**: size greater. **Head**: head yellow-gray with indistinct yellow stripes in middle, two pairs of *pas*, *pas1* situated in middle of frons, *pas2* situated at middle margin of eyes. **Thorax**: prothorax bearing a lightly pigmented tubercle, apically deeply bifurcate. Pronotum with three *as*, two *sls*, one *ds* and two *pls. As* and *sls1*, *pls* subequal, strong and long, *pls1* and *pls2* in one transverse row, *sls2* and *ds* short; Mesonotum with three *msns* on scutellum. **Abdomen**: segment I-VII with tergite bearing two setae. Spiracular area with two *ss*; **Legs**: pro-, meso- and metafemora apically bearing one pair of slightly outcurved *fes*, apex with grey circular pigmented area.

#### Specimen examined.

**CHINA: Xinjiang**: Talaticun, Qinghe (46°40.53'N; 90°27.74'E, 1285m), 28-VII-2013, *Veronica oxycarpa* Boiss., leg Chunyan Jiang (33); Hualin Park (47°51.75'N; 88°07.18'E, 886m), 5-VIII-2013, leg You Li (1).

#### Biological notes.

After collecting the host plants *Veronica oxycarpa* Boiss. with galls on 28-VII-2013, 10 larvae and 20 pupae were obtained on 1-VIII-2013.

#### Remarks.

This species has been recorded from Armenia, Bulgaria, Croatia, Greece, Iran, Iraq, Kazakhstan, Lebanon, Palestine, Slovakia, Syria, Tajikistan, Turkey, Turkmenistan and Uzbekistan. This species is a new record for China.

### Gymnetron villosipenne

Taxon classificationAnimaliaColeopteraCurculionidae

Roelofs, 1875

Gymnetron villosipenne Roelofs, 1875: 149.

#### Description.

**Adult** (Figures 7–8): sides of pronotum on basal half and base covered with dense, imbricate, broad scales; elytra with integument black to brown, interstriae covered with suberect seta-like scales arranged in one regular row; rostrum in lateral view straight, weakly narrowed from base to apex, in dorsal view slightly narrowing from antennal insertion to apex; eyes moderately convex (Caldara, 2008).

#### Specimen examined.

**CHINA: Jilin**: Dahuanggou, Antu, Yanbian (42°55.18'N; 128°37.53'E, 918m), 13-VIII-1963 (1). **Heilongjiang**: Xigou, Aihui, (49°51.45'N; 127°13.30'E, 154m), 20-VII-1963, leg Hongxing Li (1); Daxiangcun, Great Khingan (52°57.65'N; 122°31.67'E, 600m), 19-VII-1970 (1).

### Keys to *Gymnetron* adults, larvae and pupae from China

Key to adults of *Gymnetron* from China

**Table:** 

1	Metathoracic episterna covered with broad scales	**2**
–	Metathoracic episterna covered with narrow scales	***Gymnetron vittipenne***
2	Metasternum and urosternite one covered with narrow scales	**3**
–	Metasternum and urosternite one covered with broad scales	***Gymnetron miyoshii***
3	Elytra completely black	***Gymnetron villosipenne***
–	Elytra reddish with only interstria one and ten black	***Gymnetron auliense***

Key to larvae of *Gymnetron* from China (details in Table 1)

**Table:** 

1	Labrum without middle seta and sensilla, nearly all sclerotic	**2**
–	Labrum with middle seta and sensilla, partly sclerotic	***Gymnetron vittipenne***
2	Epipharyngeal setae stout, sensilla absent	***Gymnetron miyoshii***
–	Epipharyngeal setae slender, two pairs of sensilla	***Gymnetron auliense***

**Table 1.** Diagnostic features of the mature larvae of *Gymnetron* from China (Characters of *Gymnetron miyoshi* identical in *Gymnetronauliense*/*Gymnetron vittipenne* are not repeated, but indicated by “-”).

Trait	Character	*Gymnetron miyoshii*	*Gymnetron auliense*	*Gymnetron vittipenne*
**Length (mm)**		3.25–3.90	2.4–2.5	5.00–5.20
**Head**	Dorsal	*3 fs*, 1,3 absent; 4 *des*, 4 absent; 3 minute *pes*; 2 *les*; ventral 1 seta; *oc* present	-	-
	Antenna	1 *aseg*, longer than wide, with 4 *sa*	-	2 *sa*
**Mouthparts**	Mandible	2 teeth 1 flange, 2 *ms*, 1 sensillum	-	-
	Labrum	2 pairs of *cls*; 3 pairs of *lrms*; nearlly all sclerotic	-	2 pairs of *cls*, 1 pair of sensilla; 3 pairs of *lrms*, 1 *mds*; partly sclerotic
	Epipharynx	2 pairs of *als*, 3 pairs of *ams*, stout setae	2 pairs of *als*, 3 pairs of *ams*, 2 pairs of sensilla, slender setae	2 pairs of *als*, 3 pairs of *ams*, 1 pair of *mes*, stout setae
	Maxilla	*1 stps*, 2 sensilla; 2 *pfs*; 4 *vms*, short, 5 *dms*; *mxp* 2 segments, 1 *mxps*, 1 sensillum	-	3 *pfs*; 1 *mxps*, 2 sensilla
	Labium	*lbp* 1 segment, 1 sensillum; 2 *ligs*; *pmsc* “U” sclerotic, 1 *prms*, 1 sensillum; *plb* partly sclerotic, 2 *plbs*	-	-
**Thorax (one side)**	Th I	2 *prns*; 4 *dpls*; 2 *vpls*; 4 *pda* setae; 2 *msts*	4 *prns*	6 *prns*; 5 *pda* setae
	Th II	2 *prs*; 2 *pds*; 4 *as*; 1 *dpls*; 1 *vpls*; 4 *pda* setae; 2 *msts*	-	1 *prs*; 2 *pds*; 1 *dls*; 5 *pda* setae
	Th III	same as Th II	-	same as Th II
**Abdomen (one side)**	Abd I-VII	2 *prs*; 1 *pds*; 2 *ss*; 1 *dpls*; 1 *vpls*; 1 *lsts*; 3 *msts*; 7 *s*	-	1 *prs*; 2 *pds*
	Abd VIII	1 *prs*; 1 *pds*; 2 *ss*; 1 *dpls*; 1 *vpls*; 1 *lsts*; 2 *msts*	-	2 *pds*
	Abd IX	1 *prs*; 1 *pds*; 1 *pls*; 3 *sts*	-	-
	Abd X (anus)	1 seta, transverse	-	-

Key to pupae of *Gymnetron* from China (details in Table 2)

**Table:** 

1	Pronotum with one pair of apical setae, one pair of sublateral setae, two pairs of posterolateral setae and discal setae absent	**2**
–	Pronotum with three pairs of apical setae, two pairs of sublateral setae, two pairs of posterolateral setae, one pair of discal setae	***Gymnetron vittipenne***
2	Pro-, meso- and metafemora apically bearing two setae	***Gymnetron miyoshii***
–	Pro-, meso- and metafemora apically bearing one seta	***Gymnetron auliense***

**Table 2.** Diagnostic features of pupae of *Gymnetron* from China (characters of *Gymnetron miyoshii* identical in *Gymnetron auliense* are not repeated, but indicated by "-").

Traits	*Gymnetron miyoshii*	*Gymnetron auliense*	*Gymnetron vittipenne*
**Head**	1 pair of *pas*	-	2 pairs of *pas*
**Thorax (one side)**	Prothoracic tubercle shallowly split, 1 *as*, 1 *sls*, 2 *pls*; 2 *msns*; 2 *mtns*	3 *msns*; 2 *mtns*	Prothoracic tubercle deeply split, 3 *as*, 2 *sls*, 2 *pls*; 3 *msns*; 2 mtns
**Abdomen (one side)**	Abd I-VII, tergum 1 seta, 1 *ss*, 1 *dpls*, 1 *vpls*,1 *lsts*, 2 *msts*; Abd VIII, tergum with posterior tubercle, 1 seta, Sternum 2 setae; Abd IX, with *pc*; Abd X, anus transverse cleft, subterminal	-	Abd I-VII, tergum 2 setae, 2 *ss*
**Legs**	2 *fes*	1 *fes*	1 *fes*

### Biological information

**Figures 38–40.**
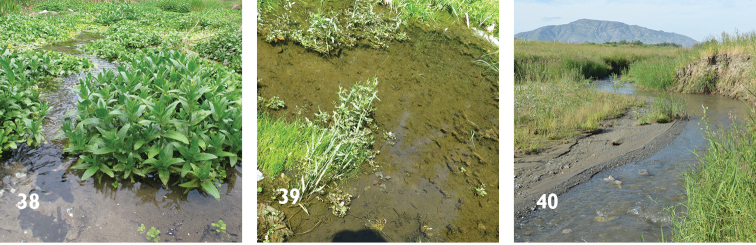
Ecological habitats of three weevils. *Gymnetron miyoshii* **39** *Gymnetron auliense* **40** *Gymnetron vittipenne*.

Host plants of *Veronica* in which larvae of the three species live are widely distributed in China (Zhong 1979). *Gymnetron auliense* and *Gymnetron vittipenne* were both collected on *Veronica oxycarpa* Boiss., *Gymnetron miyoshii* was collected on *Veronica anagallis-aquatica* L.; all host plants live on the banks or in clear slowly flowing streams (Figures 38–40).

**Figures 41–50.**
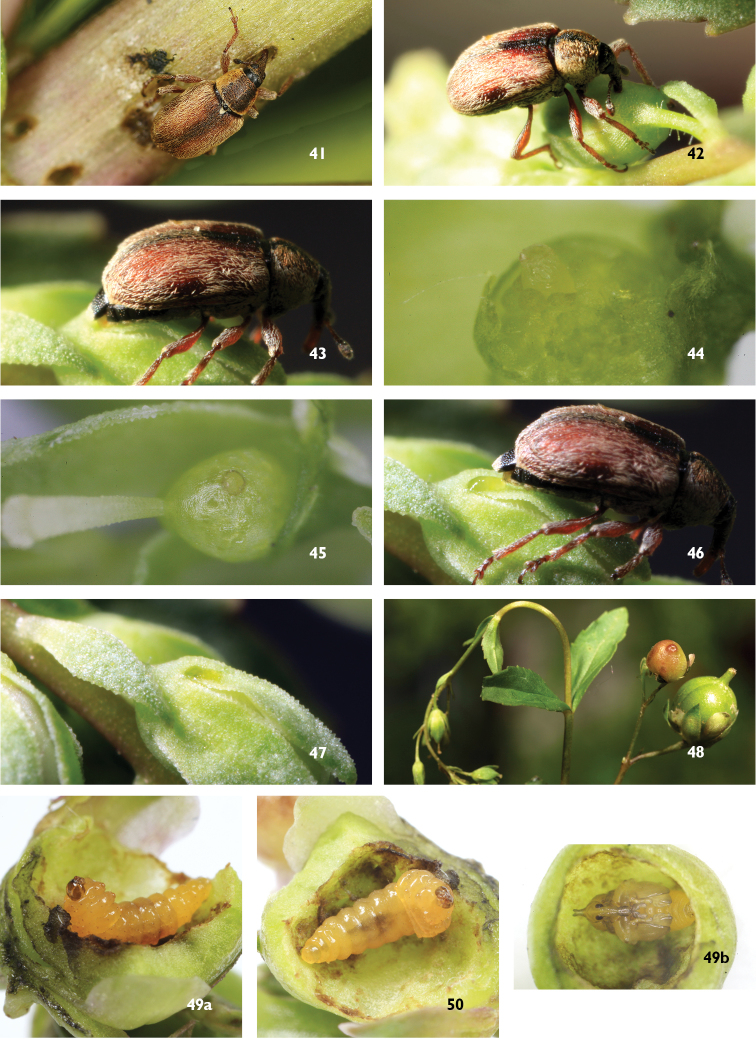
*Gymnetron miyoshii*. **41** Adult feeds on stems of *Veronica anagallis-aquatica* L. **42** Adult makes holes on bud of host plants **43** Adult lays eggs in buds **44** Egg in ovary surrounded by ovules **45** Egg visible through ovary wall **46** Adult seals the hole with fecal material **47** Dry fecal material **48** Galls of ovaries and normal flowers **49a–b** Larva **50** Pupa.

The adults of *Gymnetron miyoshii* feed on stems near the axils of their host (Figure 41). Females make holes on petals and calyces with mouthparts on the apex of the rostrum (Figure 42), and lay eggs in ovaries of developing flower buds or flowers (Figure 43). One oviposition hole can be found on the surface of one ovary and one larva develops in an ovary. The oval eggs are mostly surrounded by ovules and are evident on the ovary wall (Figure 44–45). As Howden (1995) reported, after oviposition, females seal the hole with fecal material (Figure 46–47). Larvae feed in the ovaries, stimulating ovaries to develop into galls (Figure 48). Larvae (Figure 49a–b) are active, and if disturbed, their abdomen sways front and back quickly. Mature larvae will not pupate until the ovary wall remains as a thin membrane. Pupae (Figure 50) are also active and their abdomen can sway front and back quickly like the larvae. Ovaries attacked by weevils will not produce seeds, but the damage seems not to seriously harm the plant’s whole reproductive rate. After observation in the field, we found *Gymnetron miyoshii* mostly live in the ovaries in the middle of the inflorescence, while flowers at top and bottom still produce seeds.

**Figures 51–54.**
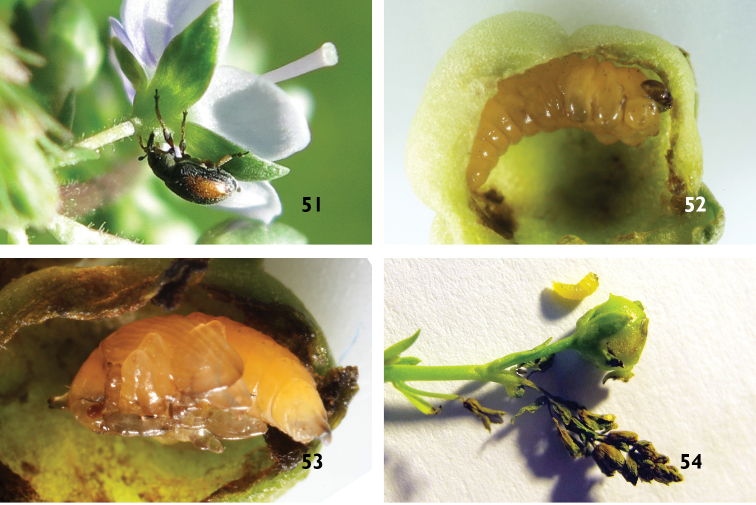
*Gymnetron auliense*. **51** Adult makes holes on bud of host plants, *Veronica oxycarpa* Boiss. **52** Larva **53** Pupa **54** Galls of ovaries and normal dead flowers.

*Gymnetron auliense*, like *Gymnetron miyoshii*, also lays eggs in ovaries of host plants (Figure 51), larvae (Figure 52) and pupae (Figure 53) live in galls of ovaries (Figure 54).

**Figures 55–58.**
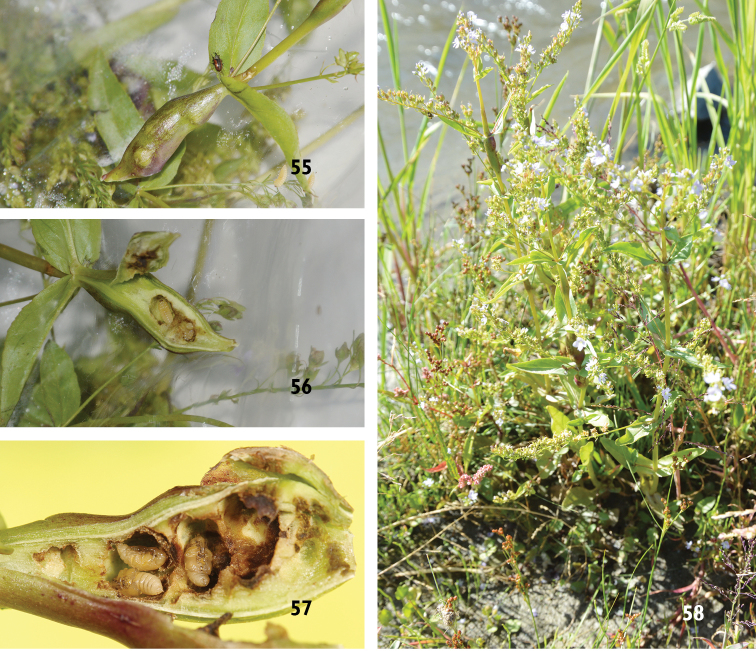
*Gymnetron vittipenne*. **55** Galls of stems **56** Larvae **57** Pupae, the two pupae on the left cell, one of them is from the right cell **58** Living host plants *Veronica oxycarpa* Boiss. with galls.

*Gymnetron vittipenne* was collected in *Veronica anagallis-aquatica* L., as reported by Ugarte San Vicente et al. (2012), where it lays eggs in stems between two leaf bases. After oviposition, galls formation occur (Figure 55). There are several cells in each gall, which are separated individually, with one larva per cell (Figure 56–57). Damage does not seriously harm the host plants, and plants keep producing flowers and seeds normally (Figure 58).

## Discussion

Lee et al. (1998) described the larva of *Gymnetron miyoshii*, but after further study, distinct major differences are observed in the head (ocelli, setae and clypeus), mouthparts (labrum, labium and maxillary palpus), setae of thorax and abdomen, and number of spiracles, with comparison in Table 3. The differences maybe due to a misidentification made by Lee et al. (1998). The character of labrum is important in dividing different group in Curculionidae. Usually labrum with one pair of sensilla is the primitive state, but with 0, 1, 3 sensillae is advanced (van Emden 1938). In our observation, there are 2 sensillae in *Gymnetron vittipenne*, but none in *Gymnetron miyoshii* or *Gymnetron auliense*, which shows the genus of *Gymnetron* may be not monophyletic. The labrum fused with the clypeus and without the epipharygeal rod is an important character of *Gymnetron*; the maxillary palpi of these 3 species are 2-segmented, but the basal segment is incomplete in *Gymnetron miyoshii* and *Gymnetron auliense*. We did not observe accessory appendages in any of the 3 species. Caldara (2013) used characters of the host plants to build the phylogenetic tree in *Mecinus* Germar, 1821. The immature stages have a close relationship with host plants. Thus, it will be a great help to add characters of immature stages in the phylogenetic studies.

**Table 3.** Different descriptions of larva of *Gymnetron miyoshii*.

Trait	Character	Description (Lee et al. 1998)	Review
**Head**	Ocellus	2 pairs	1 pair
	Setae	*des1*, *3*, *5* long, equal, *des4* short, *des2* absent; *les*, *ves* absent; 2 *pes*; 2 *cls*, different length	*des3* longest, *des5* long, *des1*, 2 short equal, *des4* absent; 2 *les*; 1 *ves*; 3 *pes*; *2 cls*, equal
**Mouthparts**	Labrum	posterior margin extended medially into clypeal zone; rods as brownish patches	posterior margin indistinct; rods absent
	Labium	3 *plbs*	2 *plbs*
**Thorax (one side)**		6 *prns*; 3 *pds*; 1 *as*; pedal lobe 2 segment, 6 setae	2 *prns*; 2 *pds*; 4 *as*; pedal lobe 1 segment, 4 setae
**Abdomen (one side)**		8 spiracles; airtube longer than diameter of peritreme; 3 folds; 1 *prs*; 2 *pds*; 1 *ss*; 2 *msts*	7 spiracles; airtube subequal as diameter of peritreme; 2 folds; 2 *prs*; 1 *pds*; 2 *ss*; 3 *msts*

In addition, setae on the alar area are found to be variable. There are usually four setae of different lengths on the alar area on each side of each larva. Five setae can be found on the alar area of the metathorax of *Gymnetron miyoshii*. Thus, the setae of the alar area are not useful diagnostic characters. There is only one middle seta on the labrum of *Gymnetron vittipenne* pupae, it is special, and we name it *mds.* The features of the larva of the genus *Gymnetron* are as follows: (1) Frontal suture not extending to mandibular membrane; (2) Antennae contiguous with frontal suture; (3) Postoccipital condyles indistinct; (4) meso-, metathorax and abdomen with two tergal folds; (5) Alar area without sclerotized or pigmented areas; (6) Spiracles bicameral; (7) Head brown with pale stripes at side and *ecl* of head; (8) Accessory sensory appendage of antenna short; (9) Anus, transverse cleft; (10) Living in galls of seeds or stems of Scrophulariaceae or Plantaginaceae.

Parasites of *Gymnetron* are few (May 1993, Gumovsky 2007), and during this study, only one parasite was found in the larvae of *Gymnetron*. Low parasitism may due to the following two reasons. First, galls can be a mechanical barrier for escape from natural enemies. Second, iridoid glycosides in host plants can help *Gymnetron* to protect it from the natural enemies. Iridoid glycosides are unpalatable and denature proteins and DNA (Bowers et al. 1986, Kim et al. 2000). Though there were none of these chemical compounds in adults (Baden et al. 2012), *Gymnetron* still can use them indirectly. Since larvae and pupae live in galls, the ovary walls with iridoid glycosides can also be a protection against invertebrate and vertebrate predators. Chinese have collected *Veronica anagallis-aquatica* L. with *Gymnetron* galls as a Chinese traditional medicine for many years, which can treat some painful and inflammatory human diseases (Zhong 1979). The main active substance in this Chinese traditional medicine is iridoid glycosides (Dong et al. 2011, Guan et al. 2011). *Gymnetron* feeds on host plants, causing the plants to produce more iridoid glycosides. Baden et al. (2012) only reported there are no iridoid glycosides in adults of *Mecinus* Germar, 1821 and *Rhinusa* Stephens, 1829. To confirm whether these chemical substances exist in larvae, pupae and adults of *Gymnetron*, further studies are needed.

While collecting these species from the field, three kinds of host plants only were found, living only in flowing water with little pollution or human disturbance. In spring, there are many host plants in the habitats, but only those with *Gymnetron* living on them can survive as they begin to flower and seed. We collected plants for rearing weevils in the laboratory, and observed the same phenomenon. Plants with galls of *Gymnetron* lasted long after seven days, but those without galls began to wilt on the second day and died on the 7th day. The host plants were reared five times under the laboratory conditions. Based on this study, we formulate the hypothesis that *Gymnetron* feed on *Veronica* causing them to produce more protective chemicals, which can help the plants to resist environmental stress. Species of *Veronica* with *Gymnetron* galls living near water cannot live in unclean polluted water for long, so we can use these two organisms as environmental indicators. So, during the co-evolution of insects and plants, there are relationships not only of plant-herbivores-predator, but herbivores and plants can also help each other to live harmoniously.

## Supplementary Material

XML Treatment for Gymnetron miyoshii

XML Treatment for Gymnetron auliense

XML Treatment for Gymnetron vittipenne

XML Treatment for Gymnetron villosipenne
